# 

**Published:** 1888-02

**Authors:** 


### CONTENTS.

ORIGINAL COMMUNICATIONS—

The Relation of Fistula in Ano and Phthisis Pulmonalis. By Joseph M. Mathews, M. D., Louisville, Ky. ..... 709
Annual Address of the President of Jefferson County, Alabama, Medical Society. By John D. S. Davis, M. D., Birmingham, Ala. ..... 715
Subperiosteal Resection of a Rib for Empyema in Children—Recovery. By Albert B. Strong, M. D., Chicago, Ill. ..... 729
Mechanical Treatment of Vomiting in Pregnancy. By J. W. Flanders, M. D., Wrightsville, Ga. ..... 733
The Relation of Sciatica to Organic Stricture of the Urethra. By J. W. Griggs, M. D., West Point, Ga. ..... 736
Hydrastis Canadensis in Uterine Troubles. By R. B. Barron, M. D., Clinton, Ga. ..... 738

SELECTIONS—

Complications of Biliary Fistula with Pregnancy ..... 741
Cholecysto-Intestinal Fistula for the Relief of Occlusion of the Common Bile Duct ..... 743

CLINIC REPORTS—

A Clinical Lecture on Mitral Regurgitation. By Nathan S. Davis, M. D., LL. D., Chicago, Ill. ..... 747

SOCIETY REPORTS—

Chicago Medical Society ..... 753

CORRESPONDENCE—

Our New York Letter ..... 763
The Cause of Milk Sickness ..... 766

BOOK REVIEWS—

Transactions of the Association of American Physicians ..... 769
Anæmia ..... 769
Massage ..... 770
Diseases of the Hair and Scalp ..... 770

EDITORIAL—

Laws Regulating the Licensing of Physicians and the Practice of Medicine ..... 771
Items ..... 772

